# Microbial Interconnections in One Health: A Critical Nexus Between Companion Animals and Human Microbiomes

**DOI:** 10.3390/microorganisms13071564

**Published:** 2025-07-03

**Authors:** Stylianos Skoufos, Elisavet Stavropoulou, Christina Tsigalou, Chrysoula (Chrysa) Voidarou

**Affiliations:** 1 Master Program in “Food, Nutrition and Microbiome”, Department of Medicine, Democritus University of Thrace, 68100 Alexandroupolis, Greece; sskoufos@uth.gr; 2 Clinic of Obstetrics and Reproduction, Faculty of Veterinary Science, University of Thessaly, 43100 Karditsa, Greece; 3 Laboratory of Hygiene and Environmental Protection, Department of Medicine, Democritus University of Thrace, Dragana, 68100 Alexandroupolis, Greece; elisabeth.stavropoulou@gmail.com (E.S.); ctsigalo@med.duth.gr (C.T.); 4 Laboratory of Animal Health, Food Hygiene and Quality, Department of Agriculture, School of Agriculture, University of Ioannina, 47100 Arta, Greece

**Keywords:** microbiota, microbiome, resistome, one health, companion animals, human

## Abstract

The One Health approach is rapidly gaining the attention of the scientific community worldwide and is expected to be a major model of scientific reasoning in the 21st century, concerning medical, veterinary and environmental issues. The basic concept of One Health, that humans, animals and their environments are parts of the same natural world affecting each other, is rooted in most ethnic as well as in many religious traditions. Despite this unity and for historical reasons, medical, veterinary and environmental sciences developed independently. The One Health concept tries to reunite these and many other relevant sciences, aiming at a deeper understanding of the interconnection between the natural world, humans and animal health. The dynamic interplay between a host’s microbiome, the microbiomes of other hosts, and environmental microbial communities profoundly influences the host health, given the essential physiological functions the microbiome performs within the organism. The biodiversity of microbiomes is broad and complex. The different areas of the skin, the upper and lower respiratory systems, the ocular cavity, the oral cavity, the gastrointestinal tract and finally the urogenital system of pets and humans alike are niches where a multitude of microorganisms indigenous and transient—commensals and pathogens, thrive in a dynamic antagonistic balance of populations of different phyla, orders, genera and species. The description of these microbiomes attempted in this article is not meant to be exhaustive but rather demonstrative of their complexity. The study of microbiomes is a necessary step towards the One Health approach to pets and humans. Yet, despite the progress made on that subject, the scientific community faces challenges, such as the limitations of studies performed, the scarcity of studies concerning the microbiomes of cats, the multitude of environmental factors affecting the results and others. The two new terms proposed in this article, the “familiome” and the “oikiome”, will aid in the One Health theoretical analysis as well as in its practical approach. The authors strongly believe that new technological breakthroughs, like Big Data Analytics and Artificial Intelligence (AI), will significantly help to overcome these hazards.

## 1. Introduction

### 1.1. One Health: A Concept with Deep Roots in History

It has been argued that the One Health concept is not new. Core ideas of One Health such as the interaction between humans, animals and the environment, can be traced in almost every aboriginal tradition as well as in most religions. The argument goes that One Health refers to the ecological–biological interconnection of all creatures on the planet and thus it is a fundamental condition for life itself. It was Hippocrates, however, who first wrote that human health can be determined by the environment (On Airs, Waters and Places), followed by Aristotle who is considered the founder of comparative pathology, by observing common traits in human and animal diseases [1,2]. In the modern era, Rudolph Virchow, one of the most outstanding medical figures of the 19th century, studying the parasite *Trichinella spiralis*, noted the similarities in the pathophysiology of many diseases affecting humans and animals [3,4]. Before him, the Italian physician and veterinarian Giovani Lancisi had already stressed the importance of the environment in the spread of diseases, while the French pioneering scientists Bourgelat, Villerme and Parent-Duchatelet founded the field of veterinary public health, establishing the health interaction between humans and animals [2]. In recent years, the term One Health appears for the first time as “One World, One Health” in the “Manhattan principles” which were declared in 2004, in a symposium of the Wildlife Conservation Society [5,6]. It was in 2010 when the notion and concept of One Health was officially globally accepted and established by the EU and UN and all other policy makers, showing that even if the concept is not entirely new, it is rapidly gaining importance [7].

### 1.2. So, What Is New About the One Health Concept?

Until recently, the discussion about the connection between human and animal health had a purely anthropocentric coordination. Humans were in the center of every health concept, and their medical needs were prioritized with respect to animals. A similar observation is valid for the relationship between ecosystems and humans. In the theory of the Exposome, humans are at the epicenter, with everything else (the specific and general environment) affecting them and not vice versa [8]. In the One Health concept there is no place for arbitrary hierarchies and priorities. Humans, animals and ecosystems are equally respected due to their biological interconnection and their impact on each other’s health. This transition of focus from one species to broader sets which include other non-human animals as well as the environment in which they live and with which they interact can be described as the transition from units to communities. Humans, their animals (pets or productive animals) and their microenvironment are the members of this community, and it is almost unthinkable that one member of the community can be healthy, and another cannot.

### 1.3. The Adventures of a Definition

So, what is One Health? Various definitions have been proposed but one must keep in mind that since One Health is involved in politics, the width and depth of a definition might affect legislation. According to the Center for Disease Control and Prevention (CDC), One Health is defined as “*a collaborative*, *multisectoral*, *and transdisciplinary approach—working at the local*, *regional*, *national*, *and global levels—with the goal of achieving optimal health outcomes recognizing the interconnection between people*, *animals*, *plants*, *and their shared environment*” [9]. The American Veterinary Medical Association considers One Health as “*the collaborative efforts of multiple disciplines working locally*, *nationally*, *and globally*, *to attain optimal health for people*, *animals and our environment*” [10]. The definition of the One Health Global Network is as follows: “One Health recognizes that the health of humans, animals and ecosystems are interconnected. It involves applying a coordinated, collaborative, multidisciplinary and cross-sectoral approach to address potential or existing risks that originate at the animal-human-ecosystems interface” [11]. The One Health institute of the Davis University of California provides a simpler definition “One Health is an approach to ensure the well-being of people, animals and the environment through collaborative problem solving—locally, nationally, and globally” [12]. When looking for an official definition, the most suitable candidate is the one which is accepted and used by the FAO/WHO and the European Union (EU): “One Health is an integrated, unifying approach that aims to sustainably balance and to optimize the health of people, animals and ecosystems” [13]. What these definitions have in common are the implications, the reactions, and the actions at the human–animal–ecosystem domain boundaries [11].

### 1.4. Humans and Companion Animals: A Long Story Made Short

Although debatable, most experts agree that the dog (*Canis lupus familiaris*) was first domesticated somewhere in northern Eurasia between 14,000 and 29,000 years ago. From the ancient gray wolves who were following the nomads and were eating hunting remains to today, where more than 360 breeds of dogs have been registered [14], and dogs have become the most widespread and the most popular domestic animal. The domestication of cats followed a different pattern. Genetic evidence suggests that felines were rather tolerated by humans 9000–11,000 years ago in the Fertile Crescent area and a second domestication wave followed 3600 years ago in Egypt, in Cyprus and elsewhere [15,16]. In EU countries, approximately 25% of households own at least one dog, raising the canine population in 2023 to around 105.35 million, while 26% of households own at least one cat, with the feline population exceeding 125 million [17,18]. This widespread cohabitation reflects not merely close contact but sustained, daily physical interactions and environmental sharing between humans and pets, creating conditions conducive to microbial exchange.

### 1.5. One Health: Humans and Canines–Felines

Dogs and cats (companion animals) share the same environment with humans, which implies not only physical cohabitation but also the exchange of microorganisms through close contact and shared surfaces [19,20]. Although humans and pets do not have identical skin or gut microbiomes, overlapping microbial taxa—particularly at the genus level—have been identified, indicating a microbial convergence due to proximity [21]. This bidirectional transmission can influence the microbiome composition and health of both hosts. The majority of the One Health literature traditionally emphasizes zoonotic threats such as rabies or vector-borne diseases like leishmaniasis and ehrlichiosis [22,23], reinforcing the view that maintaining pet health is integral to human well-being. However, framing the discussion solely around diseased versus clinically healthy animals limits the scope of the One Health paradigm, which must also consider subclinical microbial exchanges and their potential long-term effects on host immunity and microbial resilience. Equally significant are the intricate interactions within the microbial ecosystems that humans, cats, and dogs host on and within their bodies. Rather than perceiving these microbiomes as discrete entities, humans and their companion animals should be considered integral components of the broader human microenvironment, existing as interrelated subsets within the larger framework of the human macroenvironment.

This article aims to delve into the intricate and far-reaching nature of human-companion animal microbiome interactions, which have the potential to influence the lives of millions of pet owners. It discusses the short- and long-term consequences of these interactions, with a specific focus on the comparison and possible dynamic relationships between human microbiomes and the urogenital, digestive and skin microbiomes of their pets and vise versa. By examining the similarities and the differences in the microbiomes, this article seeks to illuminate their implications for health and the broader One Health framework. Furthermore, this article tries to answer the question of if the current status of knowledge is robust enough to sustain an efficient One health approach and if not to highlight the causes for this incongruity and to propose and suggest means to alleviate it.

## 2. Microbiotas, Microbiomes and a Paradigm Shift

Bacterial communities have been recognized for decades, particularly for their cooperative behavior in the gastrointestinal tract (GIT). The concept that different bacterial species work together as a functional unit has long influenced scientific thinking. Initially, these communities were referred to as “microflora,” describing populations of bacteria coexisting and interacting within specific ecological niches in humans and animals—such as the GIT, skin, respiratory tract (upper and lower) and vagina [24]. Although their exact physiological roles were not well understood at first, these microbial populations were considered integral to the organs or systems they inhabited. Over time, classical microbiological and biochemical methods gradually expanded our knowledge, revealing a broader range of species and shedding light on their ecological interactions. Early research highlighted two key functions of these communities: protecting their host environments and aiding in the breakdown of macronutrients, especially within the rumen and intestines.

The development and wide applications of the omics technologies, like the 16s rRNA sequencing and the bioinformatics, changed the landscape in a revolutionary manner. By the traditional culture techniques, less than 1% of the microorganisms of the body could be studied [25]. Dogs’ and cats’ number of microorganisms living in their GIT outnumber the ones living in the human GIT [26], while in the human body live 100 trillion microorganisms classified as 3000 species [27] encompassing 11 million genes [28]. The new tools were not just more powerful than the previous ones but exceeded the expectations advancing scientific progress at unimaginable levels. The wide biodiversity of the microorganisms was revealed, and the relations and dynamics between populations of different species were studied as well as their interactions with the host. The old terms were not sufficient anymore, so new ones have been coined.

The term microbiota refers to the complete collection of microorganisms inhabiting a specific environment [29]. These include not only bacteria, but also fungi, archaea, algae and other eukaryotes. The inclusion of viruses is debated, as they are not typically classified as microorganisms in the strict biological sense. Advances in omics technologies—particularly the sequencing of 16S and 18S rRNA genes—have enabled the detailed taxonomic classification of these organisms, from the phylum down to the species level.

Within the microbiota, the core microbiota refers to the more stable and persistent microbial members of a given environment, while the transient microbiota includes microorganisms that are only temporarily present. Importantly, transient species are not necessarily pathogens but are simply not considered part of the “physiological microbiota” [30].

This distinction is especially relevant in households where people and pets cohabit, as they share and influence each other’s microbiota [31,32]. The structure of the microbiota is dynamic and can change over time and is influenced by factors such as lifestyles, breeds, and diets [28]. However, due to its high variability, defining a universal or “normal” core microbiota remains a significant challenge [33].

The term microbiome refers to the entirety of a given habitat, encompassing not only the living microorganisms present (including the microbiota and virome) but also their collective genetic material, structural components, metabolites and the surrounding environmental conditions—such as chemical interactions with the host [34]. While some definitions restrict the microbiome to the genetic content of a specific environment—more accurately referred to as the metagenome—others use terms like the bacteriome, mycobiome and virome to specifically denote the genetic material of bacteria, fungi and viruses, respectively. In contrast to the microbiota, which focuses on the microbial populations themselves, the microbiome offers a more comprehensive view by integrating all genetic and functional aspects of the microbial ecosystem. This holistic perspective reflects the complexity and interdependence of microorganisms within their environment. These conceptual and technological advances have led to a significant paradigm shift in our understanding of microorganisms. No longer seen as passive residents on biological surfaces merely aiding host functions, microbiotas are now considered a “hidden organ” [35,36,37], rich in biodiversity and intricately involved in regulating multiple physiological processes in both animals and humans.

## 3. Pets, Owners and Their Microbiomes

This section aims to present the current knowledge on microbiomes, with a focus on highlighting their complexity rather than providing an exhaustive overview. The description is comparative in nature and is necessarily limited to bacterial species and phyla for which more data are available. While fungi, viruses, archaea, and certain eukaryotes are also integral components of microbiomes, their roles and interactions remain far less characterized and understood compared to those of bacteria.

### 3.1. Skin

Many studies emphasize that the human skin microbiome has been extensively studied, while research on the skin microbiota of dogs is more limited—and even more so for cats. This lack of data limits comparative and interaction studies. Further research is needed, and this gap should be considered when evaluating microbiome interactions between humans and pets. Table 1 presents the bacterial diversity of pet and human skin. Although the dominant bacterial phyla are similar across species, their relative abundances differ. In households where dogs are treated as family members, owners tend to have a greater skin microbiota diversity. These dogs often share similar skin microbial profiles with their owners. For example, Betaproteobacteria are commonly found on the skin of dog owners, likely transferred through close contact such as mouth-to-skin interactions, as these bacteria are typically found in the canine mouth. Overall, dog owners tend to have a more diverse skin microbiome than cat owners [28]. Pet ownership, whether of dogs or cats, influences the microbiota of specific body areas in humans. Firmicutes and Actinobacteria are dominant in the inguinal, groin, and armpit regions, while Proteobacteria dominate in the nostrils [20,38], where Corynebacterium and Staphylococcus are the most common genera [39]. Sebaceous (oily) skin areas are typically colonized by Propionibacterium, while moist areas are more commonly populated by Staphylococcus and Corynebacterium [40]. The bacterial transmission between pets and humans is bidirectional. Studies show that humans can transmit Staphylococcus aureus, including methicillin-resistant strains (MRSAs), to dogs and cats. These strains often carry antibiotic resistance genes, raising public health concerns [41,42].

### 3.2. Urinary

Table 2 presents the urogenital microbiomes of humans, dogs and cats in a comparative manner.

The microbiome of the male human genitals has not been extensively investigated. Research shows that the dominant genera differ in their abundance in the glans, prepuce and coronal sulcus [52,53,54]. However, this composition is similar to the bacteriome of adjacent anatomical sites and in adults it is related—among other factors—to their sexual contacts [54]. Similar studies for dogs and cats are not currently available to the authors’ knowledge.

Lewis et al. (2013) studied the microbiota of human urine in male and female healthy volunteers and detected nine phyla (which included 56 genera for the females, and 52 genera for males), concluding that the diversity was gender dependent and more diverse for females [55]. The same authors reported a correlation between diversity and aging for both sexes.

Coffey et al. (2023) analyzed urine samples collected from neutered dogs via cystocentesis and catheterization, identifying ten phyla. Relative abundances varied depending on the sampling method, though no significant differences were observed between genders [56]. In contrast, Burton et al. (2017) reported less microbial diversity in the urine of healthy neutered dogs [57]. In contrast, Gronsfeld et al. (2024) reported that the urine samples they analyzed were predominantly sterile, emphasizing the variability in findings across studies [58].

### 3.3. Placental Microbiomes

Although the canine placenta is of an endotheliochorial type while the human placenta is of a hemochorial type, denoting a different physiology and histology, the microbiome of the healthy placenta has been linked with successful pregnancies in both, humans and dogs. In both species the placental microbiome shows great similarities with the oral microbiome and to a lesser extent with the vaginal and the uterine microbiomes [59]. La et al. (2022) report 11 phyla and 157 genera in the placenta with significant differences between women with healthy pregnancies and women with gestation *diabetes mellitus* and other pathological conditions [60]. A possible way for bacteria to colonize and pass the placenta barrier is through bacterial ligands [61,62]. Some authors argue that the detected microorganisms could be fragments of bacteria or even killed bacteria, since the placenta is very well protected by a series of defensive cells and immune mechanisms [63]. Interestingly, the maternal side of the placenta shows a higher richness of bacteria than the fetal one [64,65,66]. Panzer et al. (2023), however, criticize the methodology of all these studies and suggest that it is not yet clear that the placenta indeed bears a microbiome and if that microbiome is commensal or infectious, leaving the subject debatable and open to future research [67].

The idea that the uterus does not carry any microorganisms during pregnancies has also been known as the “sterile womb paradigm” [63]. To test this hypothesis, Banchi et al. (2023) examined the amniotic fluid, the meconium of the newborns and the endometrium at the site of the placenta attachment, of bitches and queens during C-sections under aseptic conditions [68]. Their results show the presence of *Bacillus* spp. and *Pseudomonas* spp. in the uterus; the presence of bacillus spp. in amniotic fluid and the presence of CoN Staphylococci (*Staphylococcus epidermidis* and *Staphylococcus hominis*), *Acinetobacter baumanii* and *Acinetobacter lwoffii* in the meconium, in canine samples. The feline samples revealed the presence of *S. epidermidis* and *Pseudomonas aeruginosa* in the placenta-uterus surface, *P. aeruginosa* in amniotic fluid and *Psychrobacter sanguinis* in the meconium. Rota et al. (2021), in a similar study restricted in dams isolated *Micrococcus* spp. (*M. luteus*), *Acinetobacter* spp., *Bacillus* spp., *Demacoccus nishinomiyaensis*, *P. aeruginosa*, *Staphylococcus pseudintermedius*, *Macrococcus* spp. (*M. canis*) and *S. aureus* from the fetal surface of the placenta [69].

**Table 2:** The diversity and composition of the urogenital microbiome across humans and Companion animals.

Anatomical Site	Humans	Dogs	Cats
Male Urogenital System	Dominant genera: *Prevotella*, *Finegoldia*, *Peptoniphilus*, *Staphylococcus*, *Corynebacterium*, *Anaerococcus* [52,53,54]	No studies available.	No studies available.
Urine	Nine phyla: Acidobacteria, Actinobacteria, Bacteroidetes, Cyanobacteria, Firmicutes, Fusobacteria, Proteobacteria, Synergistetes, Tenericutes [55]	Actinobacteria, Bacteroidota, Proteobacteria, Firmicutes [56]. Dominant: Proteobacteria (*Pseudomonas*, *Sphingobium*, *Acinetobacter johnsonii*) [57]. Species: *Corynebacterium auriscanis*, *Streptococcus parasanguinis* [70]. Some found urine sterile [58]	No studies reported for cats in urine microbiome.
Placental Microbiomes	Dominant phyla: Firmicutes, Proteobacteria, Bacteroidetes, Actinobacterium [71]. Debate exists about sterile womb [63,67]	*Bacillus* spp., *Pseudomonas* spp., *Staphylococcus* spp., *Micrococcus* spp., *Acinetobacter* spp. in placenta, amniotic fluid, meconium [68,69]	*Staphylococcus epidermidis*, *Pseudomonas aeruginosa* on placenta–uterus surface, *P. aeruginosa* in amniotic fluid, *Psychrobacter sanguinis* in meconium [68]
Vaginal and Uterine Microbiomes	Vagina: Dominated by *Lactobacilli*; influenced by ethnicity, diet, stress [72,73]. Uterus: *Lactobacillus*, *Pseudomonas*, *Acinetobacter*, *Vagococcus* [74,75,76,77,78]	Vagina: Highly diverse (300+ OTUs). Dominant phyla: Bacteroidetes, Proteobacteria, Tenericutes, Firmicutes [79]. Common: *Echerichia coli*, beta-hemolytic *Streptococci*, *Staphylococci*, *Pasteurella* [80]. Uterus: *Pseudomonas*, *Staphylococcus*, *Corynebacterium* [58]	Dominant genera: *Escherichia-Shigella*, *Streptococcus*, *Pasteurella*, *Bacteroides*, *Staphylococcus* [68]. Common species: hemolytic *E. coli*, *S. canis*, *Streptococcus felis*, *Enterococcus* spp. [68]. Stage of estrous cycle, age, and body condition score did not affect diversity; domestic vs. feral environment did [68]

### 3.4. Vaginal and Uterine Microbiomes

In the healthy human vagina Lactobacilli are the dominant genus [81]. According to Holdcroft et al. (2023) the major factors affecting the vaginal microbiome are ethnicity, diet, the body mass index, psychology (mainly stress), smoking, age, the menstrual cycle (menses are associated with a decrease in *Lactobacilli* but a total increase in the biodiversity of the microbiome), contraception and pregnancy [73]. Chen et al. (2017) found a relative abundance of 97.56% Lactobacilli in the cervical mucus [74], a finding that might suggest that the presence of Lactobacilli in the endometrium could be either due to the contamination of samples or due to the contamination of the endometrium through the cervical canal [75,76]. Moosa et al. (2020) argue that the presence of Lactobacilli is affected by ethnicity and other factors [78].

The female reproductive tract in dogs represents a complex and diverse bacterial ecosystem. DNA sequencing methodologies have revealed the presence of over 300 operational taxonomic units (OTUs) within this environment [79]. This research further demonstrated that distinct bacterial communities inhabit specific anatomical regions, with the vagina and endometrium each supporting unique microbial ecosystems. These findings underscore the microbial diversity and regional specialization within the canine female reproductive tract. Gronsfeld et al. (2024) reported no significant differences in the diversity and richness of vaginal bacterial populations in the different phases of the estrous cycle [58].

Banchi et al. (2024) investigated the feline vaginal ecosystem and found that the stage of the estrous cycle, the age and the body condition score did not affect the diversity and richness of the ecosystems’ populations. However, differences were recorded with respect to the environment (domestic vs. feral cats) [68].

Stokholm et al. (2012) studied the effect of pet ownership on the vaginal microbiome of pregnant women and concluded that pets cause alterations in the vaginal microbiota and increase the risk of urinary tract infections due to *E. coli* [82].

### 3.5. Respiratory Microbiome

The microbiome of the human respiratory system has been studied in all its anatomical parts. Most authors suggest that the oropharynx is a major source for bacterial spread along the respiratory tract. Even in lungs, the most distal tissue, oropharyngeal secretions can be absorbed during sleep [83,84].

For many years, the lungs were believed to be a sterile environment. However, emerging research has identified a diverse pulmonary microbiome comprising bacterial strains. These findings have transformed our understanding of lung biology, emphasizing the lungs as a dynamic microbial ecosystem rather than a sterile organ.

In dogs, the upper respiratory system, and particularly the oropharynx, as well as inhaled air contribute to the microbiota of the lower respiratory system as they do in humans with a triple populations’ regulating mechanism of the immigration, elimination and reproduction of microorganisms [85,86]. Vangrinsven et al. (2021) report a higher abundance of *Veillonellaceae*, *Rothia*, *Pasturellaceae*, *Polynucleobacter*, *Staphylococcus cohnii*, and *Mangrovibacter* with respect to meso- and brachycephalic dogs, while no significant differences with respect to age and the environment were observed [87].

In the nasal cavity and oropharynx of a healthy cat, the most abundant phylum is Proteobacteria, while Bacteroidetes and Firmicutes are also present. The nasal microbiota is dominated by Moraxellaceae and Bradyrhizobiaceae, while in the oropharynx Pasteurellaceae, Moraxellaceae, Porphyromonadaceae, and Pseudomonadaceae are the dominant families [86,88]. Proteobacteria (Pseudomonadaceae, Sphingobacteriaceae and Bradyrhizobiaceae) are also the dominant phylum in the lungs [86,89].

Table 3 provides a comparative analysis of the respiratory microbiomes in humans, dogs and cats, highlighting shared and distinct microbial taxa that may have implications for the cross-species microbial exchange and respiratory health within the One Health framework.

### 3.6. Ocular Microbiome

Age, ethnicity, gender, lifestyle, contact lenses, ocular or systemic coexisting diseases, infections, the anatomical site of the ocular surface and pharmaceutical substances (antibiotics) are among the factors affecting the composition of the ocular microbiome in humans [100,101,102].

The canine ocular surface harbors Proteobacteria (alfa-, beta-, gamma-), Actinobacteria, Firmicutes, Bacteroidetes, and Fusobacteria although there are contradictory data about the abundance of each phylum [103,104,105].

In cats, Proteobacteria, Firmicutes, Actinobacteria, Bacteroidetes, Fusobacteria and Chlamydiae are the dominant phyla on the ocular surface [106,107]. To illustrate species-specific and shared microbial patterns, Table 4 presents a comparative analysis of the ocular microbiomes in humans and companion animals, highlighting key similarities and differences in dominant taxa.

### 3.7. Oral Microbiome

The microbiome of the human oral cavity has a complex function not only locally, but affecting the whole the organism as well. The oral cavity is a landscape with different niches due to its complex anatomy, and thus the oral microbiome shows an impressive diversity [117]. Due to differences in the abundance of certain genera in samples of the oral microbiome, various “stomatotypes” have been described. Genera *Neisseria* and *Haemophilus* belong to one stomatotype, while *Prevotella* and *Veillonella* belong to another one. There are also the “variable stomatotypes”, formed by some genera (*Streptococcus*, *Gemella*, *Porphyromonas* and *Rothia*) whose presence occurs under certain conditions and who coexist with specific bacteria [118].

The canine oral microbiome shows a broader diversity than the human one (14 phyla, 23 classes, 37 orders, 66 families and 148 genera) [119]. Flancman et al. (2018), however, report 26 phyla [120]. Ruparell et al. (2020) and Oba et al. (2021) found that the different sites in the oral cavity of the dog accommodate distinct microbiotas [121,122]. Food affects the oral microbiome since detrimental bacteria (*Fretibacterium fastidiosum*, *Filifactor alocis*, *Treponema medium*, *Tannerella forsythia*, *Porphyromonas canaris*, *Porphyromonas gingivalis*) were found to be more abundant in dogs fed wet food compared to dogs fed dry food [123]. Although bacteria from the canine oral cavity can potentially infect the dog owner’s oral cavity and cause dental diseases, their survival chances are poor due to physiological differences between these two habitats [21]. The oral microbiome’s composition is also affected by age [124].

Feline oral microbiomes have also been studied, yet various researchers’ results show a disagreement, particularly at the genus level. The breed, sex and environment significantly influence the oral microbiome of cats [51]. Table 5 provides a comparative analysis of the oral microbiomes in humans and companion animals, emphasizing both shared and distinct microbial taxa that reflect differences in their diet, physiology, and environmental exposure.

### 3.8. The Gastrointestinal Tract Microbiome

#### 3.8.1. Esophagus

In humans, the dominant phyla in the esophagus are *Proteobacteria*, *Firmicutes*, *Bacteroidetes* and *Actinobacteria*, while the dominant genera include *Streptococcus*, *Ralstonia*, *Fusobacterium*, *Neisseria*, *Haemophilus*, *Prevotella*, *Porphyromonas*, *Actinobacillus*, *Veillonella*, *Tissierella* and *Staphylococcus* [131,132,133,134]. The composition of the esophageal microbiome is significantly influenced by factors such as alcohol consumption, diet, medications (e.g., antibiotics and proton pump inhibitors), smoking, and the BMI [135].

To date, there are no published data on the esophageal microbiome of dogs and cats, and this represents a clear gap in the current veterinary microbiome research.

#### 3.8.2. Stomach

The gastric environment has been considered for a long time as hostile for microorganisms due to its acidity. Since 1982 however, when *Helicobacter pylori* was first isolated, a variety of bacteria have been detected in both the gastric mucosa and the gastric fluid. The bacterial load of the gastric environment is relatively low with respect to the intestines, estimated around 10^2^–10^4^ CFU/mL [136]. The microbiomes of the mucosa and of the gastric juice are distinct. Proteobacteria and Firmicutes dominate the mucosa, followed by *Bacteroidetes*, *Actinobacteria* and *Fusobacteria* [137], while in the gastric juice the dominant phyla are Firmicutes, Actinobacteria and Bacteroidetes [138,139]. Gastric juice may display a broader microbial diversity than the mucosa, as bacterial species like *Veillonella*, *Lactobacillus*, and *Clostridium* can migrate from the oral cavity and the duodenum and establish themselves therein [140]. *Prevotella*, *Pseudomonas*, *Streptococcus*, *Veillonella*, *Rothia*, *Helicobacter* and *Haemophilus* are the most abundant genera [141,142]. Factors affecting the gastric microbiome include age, ethnicity, gender, diet, lifestyle, medicines (antibiotics and PPI) and *H. pylori* [143,144].

As in humans, the canine and the feline stomachs carry significantly less bacterial loads (10^4^–10^5^ CFU/mL) with respect to the rest of the GI tract. *Proteobacteria* are practically the major dominant phylum with very few *Firmicutes*. Dominant genera are the *Helicobacter* spp. and the *Lactobacillus* spp. [145,146].

#### 3.8.3. The Gut

The gut microbiome is perhaps the most well studied and remains, along with the skin microbiome, the only one for which One Health claims and hypotheses can be made so far. For humans, dogs and cats, and from an anatomical point of view, the gut is divided into different sections, each one of them having a different histology and physiology with respect not only to its cellular construction but also in terms of the transit time, secretions, pH, oxygen tension and others. These differences imply that the gut microbiome is distinct in the different intestinal compartments [25]. In addition, factors such as the species, breed, age, diseases, diet, gender and genetics significantly affect the gut microbiome of companion pets. Dogs and cats are carnivores, and their gut microbiome participates in the digestion and absorption of nutrients, but it does not contribute to these processes as much as the gut microbiome of herbivores or omnivores. Yet, a healthy microbiome is an important component of many physiological processes, not only in the digestive tract but also in many more parts of the organism, due to the formation of an interaction axis with other organs, like the gut–brain axis and others [147]. It is no surprise that high interindividual variations have been observed even among pets living in the same household, thus supporting the opinion that every pet has its own unique gut microbiome [148,149].

The human gut microbiome harbors a gene pool approximately 150 times larger than that of the human genome [150]. The total amount of bacterial cells is estimated to approach one trillion with an assessed weight up to 2 kg. It is characterized as highly personalized due to the intense fluctuation of its composition among individuals [151].

Pet ownership affects the human gut microbiome. Dog owners are reported to have an increased abundance of Actinobacteria [152], while cat owners show increased populations of Proteobacteria [153]. Kates et al. (2020) argue that, although detectable, the impact of pet ownership on the human gut microbiome is not significantly important [154]. Research shows that pet dogs may marginally alter the gut microbiota of their owner [32,155,156]. Yet, pet owners tend to have a higher abundance of *Lactobacilli*, while humans without pets have a higher abundance of Streptococci and of Clostridia of the XIV cluster.

The marginal alteration of the owner’s gut microbiome by pet dogs likely results from the sustained microbial exchange via shared environments, physical contact and the exposure to pet-associated microbes. These interactions can facilitate the horizontal transfer or enrichment of specific bacterial taxa. For instance, the increased abundance of Lactobacilli in pet owners, as compared to the higher prevalence of *Streptococci* and *Clostridia* cluster XIV in non-pet owners, may reflect subtle shifts in microbial communities influenced by a cohabitation with dogs [156]. Though these changes are generally modest and vary among individuals, they suggest that close and regular contact with pets can subtly shape the human gut microbiome over time through environmental and immunological modulation [32,155,156].

- *Gut Microbiome Toxicity in the Context of One Health*

Emerging evidence highlights that environmental xenobiotics—such as heavy metals, pesticides and industrial pollutants—disrupt the structure and function of the gut microbiome, a phenomenon known as dysbiosis. This disruption has been mechanistically linked to a range of chronic conditions, including obesity, type 2 diabetes, certain cancers, immune dysfunction and reproductive disorders [157,158,159]. These toxicants interfere with microbial metabolic pathways and gene expression, thereby compromising the microbiome stability and host health [160,161]. The gut microbiome’s sensitivity to chemical exposure may represent a crucial, yet underappreciated, pathway connecting environmental contaminants to disease risk [162].

While most research has focused on humans, growing data indicate that dogs and cats—our closest companion animals—exhibit similar gut microbiome alterations associated with disease [163]. However, the specific effects of environmental xenobiotics on the gut microbiota of these species remain largely unexplored. Given that companion animals cohabitate with humans and are exposed to comparable environmental conditions, Hernandez et al. (2022) suggest that the same toxic compounds are likely to affect their gut microbiomes in analogous ways [28].

This shared vulnerability underscores the importance of integrating the gut microbiome toxicity into the One Health framework. As the environment constitutes one of its three foundational pillars, understanding how environmental chemicals modulate microbial ecosystems across species is critical. The gut microbiome, therefore, may serve as a sensitive biological interface through which environmental exposures exert parallel health impacts on both humans and animals, reinforcing the interconnectedness at the heart of the One Health concept. Table 6 summarizes the composition and diversity of gastrointestinal microbiomes in humans and companion animals, showcasing the interspecies variation in dominant microbial groups and underlining the role of host-specific factors in shaping gut microbial communities.

## 4. Can We Support the One Health Approach in Terms of Microbiomes?

The honest answer is not yet—or at least not to the extent required to fully understand the complex interactions between the microbiomes of pets and their owners, especially in terms of health and disease. Two key limitations currently hinder this effort.

The first and most significant obstacle is the absence of a clear, universally accepted definition of a “healthy microbiome.” Even in humans, whose microbiomes are among the most extensively studied, there is still no consensus on what a healthy microbiome actually looks like. To meaningfully apply the One Health concept to microbiomes in pets and their owners, we would need a robust and detailed characterization of these microbiomes as a foundational requirement. However, such a description remains elusive. Although the concept of a microbiome is well established, its specific microbial content is highly variable and dynamic. Simply put, without knowing what constitutes a healthy microbiome, it is premature to discuss the full extent of microbiome interactions across species.

As shown in earlier sections, major gaps and uncertainties remain in our knowledge. By definition, microbiomes encompass not only the microorganisms within a specific environment but also their genes, metabolites, and the ecological dynamics of their communities. Currently, our understanding is limited to a subset of the microbiota, as many operational taxonomic units (OTUs) remain unclassified, and the functions of many microorganisms are still unknown [171].

Furthermore, the imbalance in the research focus is striking: while human microbiomes have been relatively well studied, canine microbiomes have received far less attention, and feline microbiomes even less. This skewed focus contradicts the fundamental principles of the One Health framework, which aims to integrate human, animal, and environmental health in a balanced, non-anthropocentric way.

Moreover, very few studies have examined the interactions between healthy microbiomes—or the consequences of dysbiotic microbiomes—in humans and companion animals. Even fewer have explored the role of the environment and environmental microbiomes in these interconnections. In addition, while bacteria have been the primary focus of microbiome research, other essential components—such as fungi, viruses, and eukaryotic microorganisms—remain comparatively understudied, despite their critical roles in shaping microbial ecosystems.

It is clear that more comprehensive data on both human and pet microbiomes are urgently needed. One of the main obstacles in this effort is the lack of standardization across studies. Most published research to date involves small sample sizes, whether human or animal, making it theoretically questionable, if not practically impossible, to draw broad or reliable conclusions about the nature and composition of microbiomes.

Moreover, microbiomes are highly individual and subject to significant variation. This variability arises not only from biological differences between individuals (human or pet) but also from a wide array of influencing factors. For humans, these include their ethnicity, diet, gender, environment, lifestyle and age. In pets, factors such as their species, breed, age, diet, gender, environment, and physical activity also play a major role [154]. These complexities highlight the need for large-scale epidemiological studies that can provide the statistical power necessary for drawing meaningful and generalizable insights.

Another widely acknowledged limitation is the inadequacy of traditional culture-based and biochemical techniques for studying microbiomes. These methods are time-consuming and labor-intensive and fail to detect many microorganisms, particularly those that cannot be cultured in laboratory conditions. As a result, modern microbiome research relies heavily on omics technologies, particularly 16S rRNA sequencing and shotgun metagenomics. These advanced techniques offer a high sensitivity and taxonomic resolution, making them well-suited for detecting and identifying a wide range of microorganisms.

However, these methods are not without pitfalls. One issue is their extreme sensitivity, which allows them to detect even trace fragments of microbial genetic material. This can blur the line between indigenous microorganisms and transient or non-viable intruders (either commensals or pathogens). For example, in studies of the placental microbiome, some researchers argue that detected microbial DNA originates from bacteria destroyed by host immune defenses rather than from viable resident species [172]. 

A second common challenge is primer bias—a phenomenon in which certain genetic sequences amplify more efficiently than others, leading to distortions in the relative abundance of detected taxa [173,174]. A classic example of this issue is the discrepancy in findings reported by Middelbos et al. (2010) and Swanson et al. (2011), who analyzed the same canine fecal samples but arrived at different microbiome compositions due to such methodological biases [167,168].

Another limitation of high-resolution molecular techniques is their inability to provide accurate quantitative data about microbial populations within a host or environment. They are excellent at detecting presence, but are less reliable when it comes to precise microbial counts.

Despite these challenges, emerging technologies, such as machine learning and artificial intelligence, hold great promise. By using advanced training algorithms, these tools may help correct biases, distinguish between indigenous and non-indigenous microbial fragments and enhance the accuracy of microbiome analyses. We believe this is a particularly promising research avenue that should be prioritized by the scientific community.

Despite its current limitations, the One Health framework remains a powerful and promising approach, especially because it integrates humans, animals and their shared environment. This holistic perspective has already produced valuable insights, particularly in the study of the resistome, the pool of antibiotic resistance genes circulating in different environments. These genes can be exchanged between humans and pets, contributing to the formation of resistance reservoirs within their microbiomes (see relevant section).

Another very helpful idea, enhancing the One Health approach is the “core microbiota” of a microbiome, which practically divides each microbiome into the indigenous and transient microorganisms. The indigenous microorganisms, the core microbiota, are the ones with the most important physiological roles through their interaction with their host, participating in and regulating numerous biochemical, immunological and metabolic pathways [175]. The exact degree of this phenomenon is not yet fully understood, but scientific results in recent years reveal that the physiological impact of the core microbiota extends beyond the anatomical boundaries of its local niche. The transient microbiota is not necessarily pathogenic although it may include pathogenic microorganisms. Its composition reflects the dynamics of the bacterial populations of the environment, which interact with the core microbiota. To which degree crosstalk affects the physiological function of the core microbiota is unclear, although research shows that the different environments significantly affect the microbiomes of pets and humans as well.

To better understand the interactions of the microbiomes of pets, humans and the environment and to help focus the relevant research, we propose the concepts of the “familiome” and “oikiome”.

    - The familiome and the oikiome

Pets nowadays are treated as members of the family. They live in the same space with their owners, they interact with them daily, they may have similar lifestyles (especially dogs), they lick them, and, in many cases, they share the same bed and food [176]. We propose the term familiome to describe the shared microbiome of companion animals, their owners, and the household environment they inhabit. This microbiome—the familiome—serves as the common interface among the individual microbiomes and could provide valuable insight into the crosstalk between them, particularly in relation to pathogens, environmental stressors, and resistance to antimicrobial agents. The familiome could also, through its diversity, mirror the multitude of the participating microorganisms, the environmental limiting factors, its protective effect against pathogens and an assessment of dysbiotic situations. The skin, the nostrils and the oral cavity of pets and humans as well as household dust can easily provide samples for analysis. The familiome incorporates all factors which affect the individual microbiomes of pets and humans, and differences between familiomes or temporal differences in the same familiome will be useful to assess the real potency of these factors. A closely relevant concept is the “oikiome” (from the Greek word *οικία*-“oikia”-meaning the house in which people live), which refers to the microbiome of the apartment or any kind of house that a family and their pets live. The oikiome is included in the familiome but retains elements of autonomy since it can refer to an empty or a deserted house or an old house, etc.

**Figure 1:**
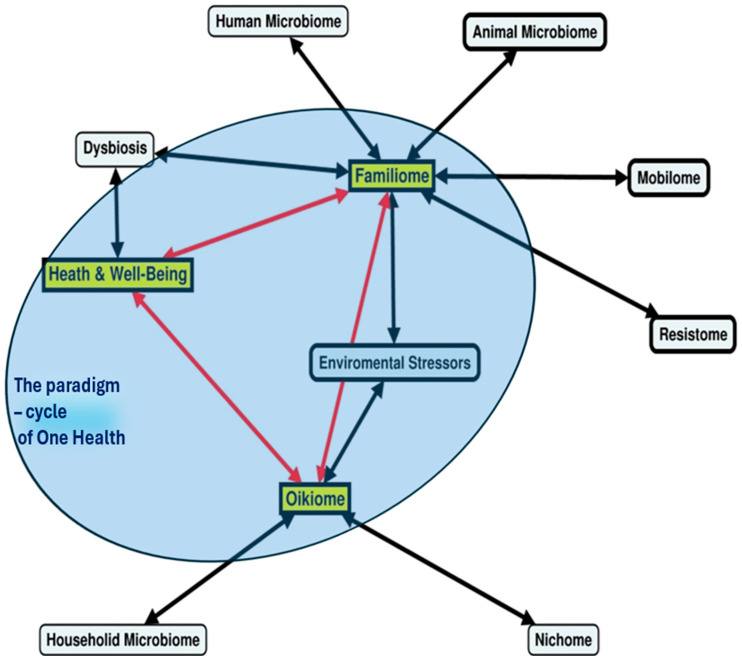
The One Health paradigm—cycle: the integration of the familiome, oikiome, and shared microbiomes in humans, companion animals and households.

One Health, one household, one “familiome” and one “Oikiome” (Figure 1): The concept of “One Health” goes beyond the traditional understanding of the interconnectedness of human, animal, and environmental health by recognizing the complex relationships within individual households, or the “familiome.” This idea highlights the profound, mutual influence between humans and their companion animals, emphasizing the deep connection that shapes health, well-being, and the shared microbiome, resistome, mobilome, and nichome within a common living environment. Understanding this connection is essential for addressing health issues that affect both humans and animals, reinforcing the holistic One Health approach at its most intimate level—within our own homes.

## 5. The Transition from the Microbiome to the Resistome

The microbiome, often described as a “superorganism”, consists of the collective community of microorganisms inhabiting specific anatomical niches and their associated genomes [175]. However, the widespread and increasing use of antibiotics has become one of the most significant factors altering the microbiome’s composition. Antibiotics not only disrupt the balance of microbial communities but also lead to changes in the pool of antibiotic resistance genes. These alterations can have profound implications for both the effectiveness of antibiotic treatments and the overall health of the human host and their pets. In today’s era of widespread antibiotic resistance—the Pan-Resistance era—the study and understanding of resistance determinants, along with the monitoring of their transmission between humans, pets, and the environment, underscore a crucial aspect of the One Health concept. The term “resistome”, introduced by D’Costa et al. in 2006, refers to the full range of antibiotic resistance genes, including those currently active, those that could become active through mutation or gene transfer and those yet to be discovered [177,178]. This concept highlights resistance as a natural aspect of microbial evolution driven by antibiotic use and underscores the need for a comprehensive approach to managing resistance that considers entire microbial communities and their genetic potential. Following its introduction, the resistome concept has become widely adopted to describe the complete set of resistance genes in any environmental or clinical sample [178]. This includes intrinsic and acquired resistance genes, as well as precursor genes (which confer mild resistance but can evolve into stronger forms) and cryptic genes (potentially resistant genes expressed at low levels or not at all under normal conditions) [179]. Also, the resistome encompasses not only antibiotic resistance genes but also those conferring resistance to antiseptics and heavy metals [180]. Despite its broad application, the concept’s implementation varies across studies, leading to inconsistencies in gene selection, methodologies and bioinformatics approaches. This variability complicates comparisons between research findings [181].

In recent decades, companion animals have significantly contributed to the physical and mental well-being of humans, leading to a notable surge in their population and an increased focus on the interactions between humans and pets. Meanwhile, many antibiotics that are not recommended for veterinary prescription in clinical practice for pets or intended for human use have been used for the treatment of pets [182,183].

**Scheme 1:**
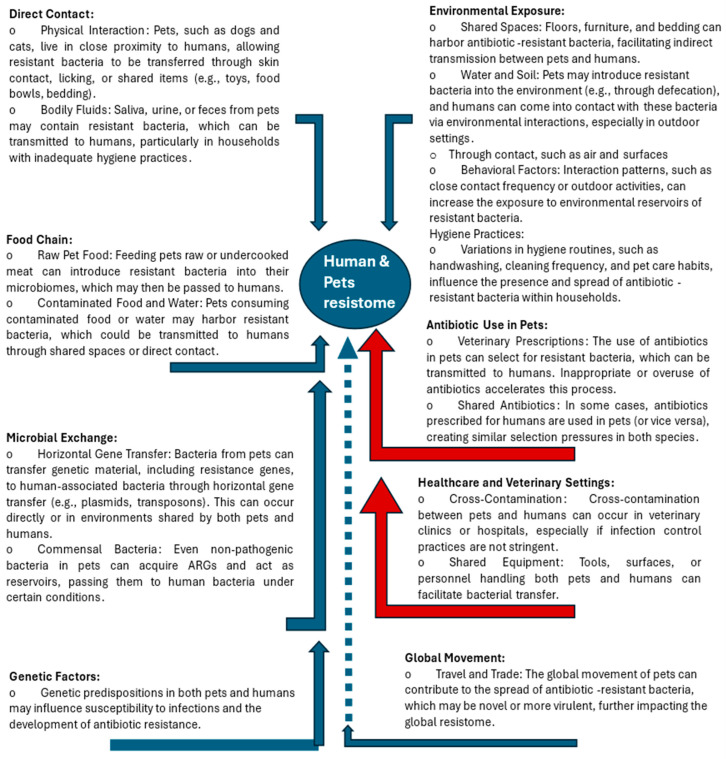
Main factors and pathways that facilitate the potential flow of antibiotic resistance genes (ARGs) between companion animals and humans: The solid red arrows represent the primary routes through which antibiotic-resistant bacteria and resistance genes flow between humans and companion animals, emphasizing the bidirectional nature of this transmission. These pathways highlight key factors such as direct contact, environmental exposure, and microbial exchange that actively shape the shared resistome. The dashed blue arrows illustrate feedback loops and amplifying mechanisms—factors like antibiotic use, healthcare settings, and global movement—that intensify resistance pressure and facilitate the persistence, evolution, and broader dissemination of antimicrobial resistance (AMR) across human-animal-environment interfaces. Together, the arrows underscore the complex and dynamic interplay that forms the core of the One Health resistome paradigm.

Today, the way humans manage and live with companion animals promotes the creation of a single ecosystem comprising humans, pets, and the microenvironment of the home. In this ecosystem (for which we propose the term “familiome”), which is often established when a pet resides permanently in the home, and specific characteristics are maintained despite minimal external influences. When discussing antibiotic-resistant genes, this can lead to the development of a robust resistome. The potential transmission of antibiotic resistance genes (ARGs) between pets and humans is a critical concern in the study of the resistome (the collection of all antibiotic resistance genes within a particular microbiome). Several factors and pathways facilitate this transmission (Scheme 1) [184,185].

Bacterial communities in humans, companion animals and their shared environments harbor extensive collections of antimicrobial resistance genes (ARGs) [177,186,187]. While significant research has focused on the resistome of livestock, the pet-associated resistome remains comparatively underexplored. Only a small subset of ARGs, typically those encountered in clinical pathogens, are well-characterized and cataloged in resistance gene databases (referred to as “established ARGs”). Recent studies have revealed that pets carry a diverse and abundant array of ARGs, highlighting their potential role as reservoirs of antimicrobial resistance [177,188,189]. Evidence from the detection of ARGs and antibiotic-resistant bacteria (ARBs) in pets and their waste further underscores this risk, suggesting that companion animals could serve as significant contributors to ARGs’ dissemination in household environments and potentially to their human owners [190,191]. These findings emphasize the need for more extensive research into pet-associated resistomes to better understand their public health implications.

When examining how pets shape the resistome of their owners, the intestinal tract emerges as the most extensively studied ecosystem. This focus is largely due to the gut’s dense and diverse microbial communities, which are major reservoirs of antimicrobial resistance genes (ARGs). Fecal samples are commonly used for analysis as they provide a comprehensive snapshot of the gut microbiota [79,192,193]. The oral cavity also garners attention because of its distinct microbial composition and susceptibility to external influences, such as antibiotics and dietary pathogens [194,195,196]. Meanwhile, the urinary system remains less explored, primarily due to its lower bacterial density and specialized microbial populations compared to5the gut and oral ecosystems [70,197]. These findings highlight the pivotal role of the gut microbiota in investigating the dynamics of antimicrobial resistance transmission between companion animals and their human owners.

Nevertheless, the interaction between companion animals and humans concerning their resistomes is a complex and evolving area of research. Identifying major resistance gene groups remains challenging due to limitations in detection methods and the need for precise analytical techniques. Recent studies focusing on the component of the two-way interaction of the resistome, with samples from the gut, highlight that the primary gene groups responsible for resistance are macrolides (*erm*B, *erm*F and broad-spectrum macrolide gene *mdf*A), beta-lactams (*bla*CTX-M, *bla*TEM, and *bla*SHV), tetracyclines (*tet*M, *tet*Q, *tet*L *mef*(A)), the non-ESBL gene, and aminoglycosides (e.g., aac and aph) that are commonly shared between humans and their companion animals. These genes are often transferred through horizontal gene transfer (HGT) mechanisms, facilitated by mobile genetic elements (MGEs), such as plasmids, integrons and transposons. For example, macrolide and tetracycline resistance genes are frequently shared between cohabiting dogs and humans, reflecting the influence of shared environments and close interaction. However, studying the frequency and direction of these transfers is challenging due to shared environmental exposures and the co-evolution of microbiomes, which add complexity to the analysis [198,199,200,201].

Although studies examining the interaction between companion animals and humans have not specifically addressed this issue, it is important to highlight an unexpected finding by researchers. The presence of the well-established tet(X4) gene, which confers a high-level resistance to tetracyclines, was identified in canine fecal matter. This is notable, as there were no previous reports of this gene being found in dogs. If the gene that confers tetracycline resistance becomes widespread, it could significantly reduce the therapeutic effectiveness of tetracyclines among pets [189,192].

A study conducted in Thailand investigated antimicrobial resistance in *E. coli* by analyzing rectal samples from healthy dogs and cats, skin and ear infection lesions, and fecal samples from veterinary staff and pet owners. The resistance genes mostly identified were bla and tet. The detection of CTX-M, TEM, and SHV gene variants confirmed the presence of ESBL-producing Enterobacteria, with *bla*CTX-M and *bla*TEM being more prevalent than *bla*SHV in all species. These results are consistent with previous studies that have reported high detection rates of these genes in Gram-negative isolates from healthcare and community settings. The growing detection of ESBL-producing *E. coli* in both healthy individuals and patients is a global concern, particularly in healthcare and community environments, with companion animals potentially serving as reservoirs for these resistant strains. The study’s high prevalence of *bla*CTX-M, *bla*TEM, and *bla*SHV highlights the potential health risks for pet owners, veterinary personnel, pets, and the surrounding environment. Additionally, the study found significant resistance related to the *tet*A and *tet*B genes, which confer tetracycline resistance through efflux mechanisms, a pattern also reported in *E. coli* isolates from humans and animals worldwide [202].

Studies examining oral cavity samples from both companion animals and their owners have identified several antimicrobial resistance genes in the microbiota of both groups. These include the CfxA family of broad-spectrum beta-lactamases, as well as genes conferring a resistance to tetracycline, streptomycin, lincomycin, pleuromutilins, and macrolides. Specifically, ribosomal protection proteins, such as tet(O), tet(Q) and tet(W), were found to confer a tetracycline resistance. The presence of these resistance genes in both dogs and their owners indicates a multispecies prevalence of antimicrobial resistance [193,194].

Abdullahi et al. 2024 studied the nasal cavities of clinically healthy dogs and their in-contact owners, finding that 23.2% of *Coagulase-Negative Staphylococci* (CoNS) isolates from healthy dog owners tested positive for the *mecA* gene, which is carried by various *SCCmec* elements. In contrast, less than 20% of clinically healthy humans tested positive for this gene. Additionally, half of the CoNS isolates from both dogs and their owners were resistant to penicillin—which is mediated by the *blaZ* gene, which produces beta-lactamase—a rate like those reported in other studies. This penicillin resistance is not unexpected given the widespread use of the antibiotic. Over 50% of the CoNS isolates exhibited resistance to macrolides, lincosamides, and streptogramins (MLS), displaying resistance phenotypes/genotypes such as erythromycin-resistant–clindamycin-susceptible and erythromycin–clindamycin-constitutive. These resistance patterns are concerning due to the importance of these antibiotics in treating staphylococcal infections. Notably, the detection of the *erm*(*T*) gene in *S. epidermidis* and *S. hominis* suggests the emergence of a new mechanism of erythromycin–clindamycin-constitutive resistance in both humans and animals [203].

Finally, studies have shown that antibiotic resistance genes (ARGs) in environments like manure can become airborne and spread through particles in residential areas where companion animals and people live. To assess the risk of aerosolized antibiotic-resistant bacteria (ARBs) in pet hospitals, researchers collected air samples from five pet hospitals [204]. This study aimed to evaluate the potential risk to hospital personnel and to inform strategies for controlling the generation and spread of antibiotic-resistant microbial aerosols in these settings.

The transposon gene tnpA-07 exhibited the highest relative expression. The top ten most expressed genes included two transposons, three tetracycline-resistance genes, two MLSB (Macrolides, Lincosamides, and Streptogramins B) genes, and three aminoglycoside resistance genes. The genes detected were associated with the resistance to various antibiotics, such as carbapenems, penicillins, cephalosporins, quinolones, aminoglycosides, macrolides and vancomycin. The relative expression of these genes is summarized, with mobile genetic elements (MGEs) showing the highest expression at 43.72%, followed by resistance to aminoglycosides, tetracyclines, MLSB, β-lactamsn, and fluoroquinolones, among others. The frequency of the expression for efflux genes was low at only 0.66%, and vancomycin resistance genes had a relatively low expression level (5.77%). The study detected 35 MLSB genes, 31 tetracycline genes, 27 β-lactam and aminoglycoside genes, 20 fluoroquinolone-associated genes, 10 vancomycin genes, 7 MGEs, 3 sulfonamide genes, and 5 other resistance-associated genes. The findings highlight a significant presence of antibiotic-resistant bacteria (ARBs) in air samples from pet hospitals, with the potential to enter the lower respiratory tract. These ARBs exhibit high levels of antimicrobial resistance, with many showing multidrug resistance (MDR). Additionally, mobile genetic elements (MGEs) are notably more expressed in these isolates. Given that ARBs in particulate matter (PM_2.5_) aerosols increase the risk of antimicrobial resistance (AMR) transmission and infection in humans, it is crucial to implement stringent controls on the use of antibacterial antibiotics to mitigate this risk.

Most studies highlight the bidirectional transmission of antimicrobial resistance (AMR) bacteria between humans and pets, as a critical and growing area of research [192,205]. While the subject has garnered significant attention in the past two decades, the evidence supporting this phenomenon often lacks robustness. The majority of studies reviewed inferred transmission based on circumstantial or indirect findings rather than direct evidence. To confirm such transmission, a more rigorous methodology is needed, involving culturing isolates from both humans and pets, conducting standardized susceptibility tests and performing detailed strain typing to establish genetic relatedness.

The reliance on case reports or case series in this field underscores the need for better research frameworks. Longitudinal studies, cohort analyses, or case–control designs would provide stronger evidence by assessing the dynamics of bacterial transmission over time and under varying conditions. These approaches could also better identify specific risk factors or circumstances that facilitate AMR transmission. Interestingly, some studies have minimized the concern over resistance gene transmission between pets and their owners, attributing it to a low antimicrobial selection pressure [192]. This perspective suggests that without significant selective pressure, such as the overuse or misuse of antibiotics, the risk of gene transfer may remain limited. However, this stance could be overly optimistic, as even low levels of selection pressure can drive resistance over time, especially in close-contact environments like households. In conclusion, while the current body of research acknowledges the potential for AMR transmission between pets and humans, it is hampered by methodological limitations. Addressing these gaps with more rigorous studies will be critical for fully understanding the risks and informing appropriate interventions.

Next-generation sequencing (NGS) has emerged as a transformative tool in resistome research, offering several key benefits such as a comprehensive detection, high-resolution comparisons, strain typing and phylogenetics, tracking mobile genetic elements (MGEs) and, finally, metagenomic insights [206,207,208,209].

The application of next-generation sequencing (NGS) in evaluating resistome interactions between companion animals and their owners highlights its transformative potential. By providing comprehensive data on the presence, mobility, and transmission dynamics of antibiotic resistance genes (ARGs), NGS enables targeted interventions to mitigate antimicrobial resistance (AMR) risks in shared environments. These insights are crucial for understanding the transfer of ARGs between humans, animals and their surroundings, forming the foundation for effective control strategies. Despite its promise, integrating NGS into routine resistome risk assessments faces significant challenges. Key obstacles include logistical requirements such as specialized equipment and expertise, the complexity of interpreting large datasets, and the high costs associated with the technology. Addressing these barriers is essential to fully harness NGS in the fight against AMR. Encouragingly, rapid advancements in bioinformatics and functional metagenomic techniques have greatly enhanced the identification and characterization of ARGs. These methods have overcome limitations associated with uncultivable microorganisms, allowing researchers to link ARG genotypes to their phenotypic expressions. This progress has facilitated the discovery of novel ARGs directly from metagenomic samples, driving innovation in the global effort to combat antimicrobial resistance.

## 6. Summary and Outlooks—One Health Approach for Pets and Humans

Given the critical biological functions of microbiomes and the prominent role pets play in modern households, the One Health approach is the most appropriate strategy for addressing and preventing health issues of both human and animal concern. However, the current knowledge remains insufficient to fully support One Health initiatives. Consequently, microbiome research must intensify and progress in a unified direction to counteract the hazards already documented.

**Expanded Research Protocols**: Robust studies involving larger populations of pets, humans, and their environments are essential to define the healthy core microbiota and microbiome. Such research should encompass not only bacteria but also fungi, viruses, and eukaryotes.

**Standardization of Methodology**: A consensus on research methodologies and techniques is vital to ensure the comparability of results across studies.

**Core–Transient Microbiome Interaction**: Investigating the interaction between the core and transient microbiomes is crucial. The transient microbiome, often originating from the environment, may influence the physiological functions of the core microbiome in ways that remain poorly understood.

**New Terminology—*Familiome* and *Oikiome***: We propose the term *familiome* to describe the shared microbiome among humans, pets, and their household environment. For the household-specific microbiome, we introduce the term *oikiome*. These concepts provide valuable tools for theoretical exploration and practical application in microbiome studies.

**Environmental Determinants and Transient Microbiome**: The transient microbiome, including its pathogenic components, must be analyzed in relation to environmental factors. While these determinants are numerous, categorizing and analyzing them could uncover patterns. Examples include tracking daily pet-owner interaction times (e.g., 1–3 h, 4–6 h), the number and species of pets, the age and number of children in the household, pet food quality, and feeding routines.

**The Significance of the Resistome**: The resistome is of paramount importance due to its role in antimicrobial resistance (AMR). The bidirectional dissemination of antibiotic resistance genes (ARGs) between pets and humans emphasizes the need for antibiotic prescriptions to consider the existing resistome within the *familiome*.

Big Data and AI technologies are expected to have a transformative role in microbiome research. These tools will enable a deeper insight into the physiological roles of microbiomes in health and disease, fostering an improved symbiosis between humans and pets while advancing our understanding of microbiome science.

## Figures and Tables

**Table 1:** Comparative overview of skin Mmicrobiomes in humans, dogs, and cats.

	Dominant Phyla	Common Families/Genera	Factors Influencing Diversity	Observations	References
Humans	Actinobacteria, Firmicutes, Proteobacteria, Bacteroidetes	Coagulase negative *Staphylococci*, *Cutibacterium* spp., *Corynebacterium* spp., *Micrococcus* spp., *Streptococcus* spp., *Acinetobacter* spp.	Skin location, moisture	Colonized by 10^3^–10^4^ CFU/cm^2^ of bacteria; 16 phyla identified; fungal infections rising among pet owners due to close contact	[19,43,44,45,46,47,48,49]
Dogs	Proteobacteria, Oxalobacteriaceae, Fusobacteria, Actinobacteria, Bacteroidetes, Firmicutes, Tenericutes, Cyanobacteria	Porphyromonadaceae, Moraxellaceae, Pasteurellaceae, Pseudomonadaceae	Skin region, gender, body site, physiology, individual factors	Diversity similar to human microbiota in families; mouth-to-skin transmission of Betaproteobacteria observed	[26,28,50]
Cats	Proteobacteria, Firmicutes, Actinobacteria, Bacteroidetes	-	Less biodiversity compared to dogs	Limited comparison to canine microbiota	[51]

**Table 3:** Comparative analysis of respiratory microbiomes in humans and companion animals.

Section	Humans	Dogs	Cats
Upper Respiratory Microbiome	Oropharynx is major source of bacterial spread; Actinobacteria (*Corynebacterium*, *Propionibacterium*), Firmicutes (*Streptococcus* in children, *Staphylococcus* in adults), Bacteroidetes (low density), Moraxellaceae (children), Gamma-proteobacteria (adults) [90,91,92,93,94]	Upper respiratory system and inhaled air influence lower respiratory microbiome; nasal cavity: Proteobacteria, Bacteroidetes, Firmicutes, Tenericutes [94,95]. Proteobacteria dominant in oropharynx and lower respiratory tract; Pasteurellaceae, Moraxellaceae, Pseudomonaceae [86].	Nasal cavity and oropharynx: Proteobacteria, Bacteroidetes, Firmicutes; nasal: Moraxellaceae, Bradyrhizobiaceae; oropharynx: Pasteurellaceae, Moraxellaceae, Porphyromonadaceae, Pseudomonadaceae; lungs: Proteobacteria dominant [88,96]
Lung Microbiome	*Prevotella*, *Sphingomonas*, *Pseudomonas*, *Acinetobacter*, *Fusobacterium*, *Megasphaera*, *Veillonella*, *Staphylococcus*, *Streptococcus*, *Porphyromonas*, *Haemophilus*; fungi: *Aspergillus*, *Cladosporium*, *Penicillium*; Enterobacteriaceae; *Tropheryma whipplei* [92,97,98,99]	Pulmonary microbiota: dominated by Pseudomonaceae and Moraxellaceae; *Cutibacterium*, *Streptococcus*, *Acinetobacter*, *Pseudomonas* abundant in lower airways [86]	Proteobacteria (Pseudomonadaceae, Sphingobacteriaceae, Bradyrhizobiaceae) dominant [86,96]

**Table 4:** Comparative analysis of ocular microbiomes in humans and companion animals.

Anatomical Site	Humans	Dogs	Cats
Conjunctiva and cornea	Proteobacteria, Firmicutes, Actinobacteria, Bacteroidetes and Deinococcus-Thermes are the dominant phyla [102,108]. *Pseudomonas*, *Propionibacterium*, *Bradyrhizobium*, *Corynebacterium*, *Acinetobacter*, *Brevundimonas*, *Staphylococcus*, *Aquabacterium*, *Sphingomonas*, *Streptococcus*, *Streptophyta*, *Ralstonia*, *Anaerococcus*, *Finegoldia*, *Simonsiella*, *Veillonella*, *Milisia*, *Massilia*, *Rothia*, *Neisseria* and *Methylobacterium* [101,105,106,107,108,109,110,111,112,113,114,115].	Proteobacteria (alfa-, beta-,gamma-), Actinobacteria, Firmicutes, Bacteroidetes, Fusobacteria. Dominant families: Pseudomonadaceae, Micrococcaceae, Pasteurellaceae, Microbacteriaceae, Enterobacteriaceae, Neisseriaceae, Moraxellaceae, Bifidobacteriaceae, Lachnospiraceae and Corynebacteriaceae [103,104,105]	Proteobacteria, Firmicutes, Actinobacteria, Bacteroidetes, Fusobacteria and Chlamydiae [106,107]. Corynebacteriaceae, Helicobacteraceae, Moraxellaceae and Comamonadaceae are the most common families [106]. *Mycoplasma*, *Streptococcus*, *Pseudomonas Staphylococcus felis* and *Moraxella osloensis* are often isolated from the ocular surface of cats [107,116]

**Table 5:** Comparative analysis of oral microbiomes in humans and companion animals.

Species	Phyla	Dominant Families/Genera	Study	Notes
Human	Actinobacteria, Bacteroidetes, Firmicutes, Proteobacteria	*Actinomyces*, *Atopobium*, *Corynebacterium*, *Rothia*, *Campylobacter*, *Cardiobacterium*, *Haemophilus*, *Neisseria*, *Fusobacterium*, *Streptococcus*, *Veilonella*, *Prevotella*	[117,125]	Describes stomatotypes based on genus abundance.
Canine	Firmicutes, Proteobacteria, Bacteroidetes, Spirochaetes, Synergistetes, Actinobacteria, Fusobacteria, Tenericutes	-	[119]	Distinct microbiota in different oral sites; diet affects composition.
Canine	26 phyla, mainly: Actinobacteria, Bacteroidetes, Firmicutes, Proteobacteria, Spirochaetes	-	[120]	
Canine	-	*Fretibacterium fastidiosum*, *Filifactor alocis*, *Treponema medium*, *Tannerella forsythia*, *Porphyromonas canaris*, *Porphyromonas gingivalis*	[123]	Detrimental bacteria more abundant in dogs fed wet food.
Feline	18 phyla	Proteobacteria, Bacteroidetes, Spirochaetes, Fusobacteria, Firmicutes, Actinobacteria	[126]	Most abundant genera include Pasteurellaceae, Moraxella, Thermomonas, Comamonadaceae and Neisseria.
Feline	Bacteroidetes, Firmicutes, Proteobacteria	-	[127]	Most abundant genera include Porphyromonas, Treponema and Fusibacter.
Feline	-	*Streptococcus*, *Staphylococcus*, *Neisseria*, *Pasteurella*	[128]	
Feline	-	*Porphyromonas gulae*, *Porphyromonas circumdentaria*, *Moraxella* spp., *Bacteroidales* spp.	[129]	Detected 249 bacterial genera and 186 genera of fungi; most prevalent fungus was *Sacharomyces cerevisiae.*
Feline	-	*Flavobacterium, Moraxella, Conchiformibius, Neisseria, Bergeyella, Streptococcus, Catonella, Actinobacillus*	[130]	Influenced by breed, sex, and environment.

**Table 6:** Diversity and distribution of gastrointestinal microbiomes in humans and companion animals.

Section/ Samples	Species	Dominant Phyla	Dominant Genera	Factors Affecting Microbiome	References
Esophagus	Humans	Proteobacteria, Firmicutes, Bacteroidetes, Actinobacteria	*Streptococcus*, *Ralstonia*, *Fusobacterium*, *Neisseria*, *Haemophilus*, *Prevotella*, *Porphyromonas*, *Actinobacillus*, *Veillonella*, *Tissierella*, *Staphylococcus*	Alcohol consumption, diet, medicines, smoking, BMI	[131,132,133,134]
Stomach	Humans	Proteobacteria, Firmicutes, Bacteroidetes, Actinobacteria, Fusobacteria	*Helicobacter*, *Prevotella*, *Pseudomonas*, *Streptococcus*, *Veillonella*, *Rothia*, *Haemophilus*	Age, ethnicity, gender, diet, lifestyle, medicines, H. pylori	[19,137,141,142,143,144]
Stomach	Dogs	Proteobacteria	*Helicobacter* spp., *Lactobacillus* spp.	Less bacterial load compared to intestines	[145,146]
Gut	Humans	Bacteroidetes, Firmicutes	*Escherichia coli*, *Klebsiella*, *Enterococcus*, *Bacteroides*, *Ruminococcus*, *Dorea*	Species, breed, age, diseases, diet, gender, genetics	[25,148]
Gut	Cats	Firmicutes, Proteobacteria, Bacteroidetes, Fusobacteria, Actinobacteria	*Clostridiales*, *Lactobacilli*, *Bacteroidales*	Species, breed, age, diseases, diet, gender, genetics	[25,164]
Gut	Dogs	Firmicutes	*Clostridiales*, *Lactobacilli*, *Enterobacteriales*	Species, breed, age, diseases, diet, gender, genetics	[26,165,166]
Fecal Samples	Dogs	Firmicutes, Bacteroidetes, Actinobacteria, Proteobacteria	*Bacteroides*, *Bifidobacterium*, *Clostridium*, *Lactobacillus*, *Enterococcus*	Methodology differences affect species detection	[167,168]
Fecal Samples	Cats	Firmicutes, Actinobacteria	Proteobacteria, Bacteroidetes, Fusobacteria	Overall population distribution in healthy cats	[169,170]

## Data Availability

No new data were created or analyzed in this study.
